# Effectiveness of Simulation‐Based Teaching Methods in Learning Obstetric Emergencies Among Healthcare Students: A Systematic Review and Meta‐Analysis

**DOI:** 10.1155/ogi/7517075

**Published:** 2026-06-09

**Authors:** Lídia Lima Aragão Sampaio, Leila Souza Brito Santos Oliveira, Murilo Araújo Oliveira, Victor Hugo de Oliveira Ribeiro, Vinícius Louzada Castro, Naiara Fonseca de Souza, Larissa Teles Martins, Vinícius Raimundo Santos da Silva, Eduardo Silva Reis Barreto, Elvis Paim Ferreira, Gabriel Souza Medrado Nunes, Renata Lopes Britto

**Affiliations:** ^1^ Federal University of Bahia, Salvador, Bahia, Brazil, ufba.br; ^2^ University of São Paulo, São Paulo, Brazil, usp.br

**Keywords:** clinical competence, knowledge, medical education, obstetric emergencies, simulation training

## Abstract

**Objective:**

To assess whether simulation‐based education improves learning outcomes in obstetric emergency management among healthcare students compared with traditional teaching methods.

**Data Sources:**

PubMed, Embase, Global Index Medicus, and Web of Science were searched from database inception to September 2025 using controlled vocabulary and free‐text terms related to healthcare students and obstetric emergencies. Only randomized controlled trials published in English were included.

**Methods of Study Selection:**

Randomized controlled trials comparing simulation‐based training with nonsimulation educational methods among healthcare students were eligible. Two reviewers independently screened titles, abstracts, and full texts using Rayyan software, with disagreements resolved by a third reviewer. Nine studies met the inclusion criteria.

**Tabulation, Integration, and Results:**

Data were extracted independently using a standardized form, and risk of bias was assessed with the Cochrane RoB 2 tool. Standardized mean differences were pooled using a random‐effects model with restricted maximum likelihood estimation. Seven studies (*n* = 333) were included in the meta‐analysis. Simulation‐based training significantly improved post‐test knowledge compared with conventional methods (SMD 0.84; 95% CI 0.36–1.32; prediction interval −0.36 to 2.05; *I* ^2^ = 75.1%), corresponding to an estimated increase of 8.4 points on the Knowledge Assessment Form (95% CI 3.6–13.2). Additional trials reported improvements in teamwork and critical thinking.

**Conclusion:**

Simulation‐based education was associated with greater knowledge gains than traditional teaching methods in obstetric emergency training among healthcare students. However, substantial heterogeneity and a prediction interval that crosses the null suggest that its effectiveness may vary across contexts. Further research should evaluate long‐term retention and clinical outcomes.

## 1. Introduction

Maternal morbidity and mortality remain a global public health challenge, particularly in the context of obstetric emergencies. Among the main direct causes are hypertensive disorders of pregnancy, such as preeclampsia and eclampsia, and obstetric hemorrhage, especially postpartum hemorrhage resulting from uterine atony, which accounts for up to 70% of cases [1]. The involvement of multidisciplinary teams is considered essential to reducing adverse outcomes, as coordination among obstetricians, nurses, anesthesiologists, and neonatologists promotes early detection, rapid decision‐making, and the implementation of effective interventions [2].

Clinical simulation, as an active teaching–learning methodology, has been increasingly employed in the training and professional development of healthcare practitioners. It serves as a complementary strategy to traditional education and is applied in undergraduate and graduate programs in nursing, medicine, and physiotherapy, as well as in continuing professional education initiatives [3]. Evidence indicates that simulation enhances the acquisition of technical and psychomotor skills, bridging the gap between theory and practice, and contributes to greater safety and confidence among professionals when performing procedures [4, 5].

In the obstetric field, simulation‐based training for emergencies has demonstrated consistent benefits. Studies have shown its effectiveness in the management of shoulder dystocia and umbilical cord prolapse, improving both technical competencies and clinical outcomes [6]. Other reviews have highlighted reductions in errors, improved mastery of techniques, enhanced team communication, and more favorable neonatal outcomes [7]. Specifically, simulation training has been associated with faster and more effective execution of critical maneuvers during emergencies, positively influencing neonatal results [8].

Despite these advances, gaps remain in the literature regarding the use of simulation for team training in obstetric emergencies. Most available studies focus on immediate improvements in technical and nontechnical skills; however, few have robustly investigated its impact on maternal and neonatal clinical outcomes [8]. In this context, the present systematic review aims to evaluate whether team‐based simulation training for obstetric emergencies, compared with traditional methods or no training, improves the performance of technical skills.

## 2. Methods

This is a systematic literature review with meta‐analysis, conducted in accordance with the Preferred Reporting Items for Systematic Reviews and Meta‐Analyses (PRISMA) [9] guidelines and based on the methodological recommendations of the Cochrane Handbook of Systematic Reviews of Interventions [10], in order to ensure transparency, reproducibility, and scientific rigor. The objective of this review is to determine whether simulation‐based teaching methods are more effective than conventional or nonsimulation approaches in improving learning outcomes related to obstetric emergencies among healthcare students.

The research question was structured according to the PICO framework, in which the Population consists of healthcare students enrolled in medicine, nursing, or midwifery programs at undergraduate or postgraduate levels who do not yet hold professional licensure in obstetric care; the Intervention refers to simulation‐based educational strategies designed to teach the management of obstetric emergencies (such as postpartum hemorrhage, eclampsia, shoulder dystocia, and umbilical cord prolapse), using high‐, medium‐, or low‐fidelity manikins, task trainers, standardized patients, or virtual/hybrid models; the Comparator comprises conventional educational approaches without hands‐on simulation, including lectures, e‐learning modules, or protocol‐based instruction; and the Outcomes of interest encompass learners’ performance in knowledge acquisition, technical and nontechnical skills, confidence, and self‐efficacy during obstetric emergency training. The review protocol was developed and registered in the International Prospective Register of Systematic Reviews (PROSPERO; registration number CRD420251117301) prior to the commencement of the database search.

### 2.1. Eligibility Criteria

Studies were eligible for inclusion if they investigated the effectiveness of simulation‐based educational interventions for teaching obstetric emergencies to healthcare students. No restrictions were applied regarding participants’ sex, ethnicity, geographic setting, or academic context. Eligible studies compared at least one simulation‐based training modality with conventional teaching approaches lacking hands‐on simulation.

Only randomized controlled trials (parallel‐group or cluster RCTs) comparing simulation‐based interventions with nonsimulation control conditions were included. Studies were excluded if they involved practicing healthcare professionals without student status, if they addressed nonemergency obstetric scenarios (such as normal labor or prenatal counseling), or if they lacked active hands‐on simulation components. Additionally, nonrandomized studies, observational studies, case reports, case series, narrative reviews, systematic reviews, meta‐analyses, editorials, commentaries, letters to the editor, conference abstracts, or studies without a comparator group or sufficient data for quantitative synthesis were excluded.

### 2.2. Databases and Search Strategy

Electronic searches were conducted in PubMed, Embase, Global Index Medicus, and Web of Science. Studies published in English were included, covering the period from database inception to September 2025. The search strategy combined controlled descriptors from the specific vocabularies of each database: MeSH terms (via https://www.ncbi.nlm.nih.gov/mesh) were used for PubMed, Global Index Medicus, and Web of Science, while Emtree terms (via https://www.embase.com/emtree) were applied to Embase. Additional nonstandard descriptors were also used to refine the strategy as the pilot search was adapted. The search terms included concepts related to students and obstetric emergencies, which were combined using Boolean operators (AND, OR). The full search strategy for each database is available in Supporting Information 1.

### 2.3. Selection of Studies

Two reviewers (GSMN and EPF), who had no access to each other’s assessments, evaluated the identified article titles, selecting those that met the inclusion criteria. Subsequently, abstracts were reviewed for selection purposes. Articles that passed the initial screening were examined in their entirety to determine inclusion in the systematic review. In situations where discrepancies arose between the two reviewers, a senior reviewer (LLAS), who had exclusive access to the conflicting articles, intervened to resolve differences. The selection of the studies was made using the Rayyan app [11]. To ensure comprehensive coverage of the available literature, a snowballing strategy [12] was also employed: Reference lists of all relevant reviews identified through the initial search were screened to identify additional eligible primary studies, and the references of the included full‐text articles were likewise reviewed after complete reading to detect any studies potentially overlooked in the database search.

### 2.4. Data Summarization

Data extraction was performed independently and in duplicate (GSMN and VRSS) to ensure accuracy and reliability. A standardized data extraction form, predesigned in Microsoft Excel (Version 2205), was used to record relevant study characteristics. Extracted information included details on the study, study design, simulation modality, comparator characteristics, outcome measures assessed, instruments or scales used for evaluation, duration and frequency of the intervention, and other pertinent methodological aspects. Any discrepancies between the two reviewers were resolved through consultation with a senior reviewer (LLAS) to achieve consensus.

### 2.5. Quality Assessment

The risk‐of‐bias assessment was conducted using the Cochrane Risk of Bias tool for randomized trials (RoB 2) [13], evaluated independently by two reviewers (VRSS and ESRB), with discrepancies resolved through consultation with a senior reviewer (LLAS) to achieve consensus. Publication bias was investigated by contour‐enhanced funnel plot analysis of point estimates about study weights [14].

### 2.6. Statistical Analysis

Because the included studies used different measurement instruments and outcome scales to assess learning performance, results were synthesized using the standardized mean difference (SMD) as the summary measure of effect. For post‐test knowledge, the pooled SMD was back‐transformed to the Knowledge Assessment Form (KAF) scale by multiplying the SMD by a representative standard deviation from that scale. If studies reported medians and interquartile ranges (IQRs) or medians and ranges, these values were converted into means and standard deviations using the methods described by Luo et al. [15] and Wan et al. [16].

All outcomes were presented with 95% confidence intervals (CIs). The Cochran *Q* test and *I* ^2^ statistics were used to assess heterogeneity, with *p*‐values < 0.10 and *I* ^2^ > 25% considered significant for heterogeneity. Given the expected methodological variability, a random‐effects model was applied regardless of the heterogeneity statistics as a conservative approach to synthesizing results, employing the restricted maximum likelihood (REML) method. When Tau [2] was different from zero, we used the Hartung–Knapp–Sidik–Jonkman (HKSJ) adjustment to estimate CIs [17]. To address heterogeneity and estimate the expected effect size in future studies, we calculated prediction intervals (PI), which were reported to account for between‐study variability, reflecting the expected range of effects in future studies [18].

No subgroup analyses or meta‐regression were performed due to the limited number of studies included in the meta‐analysis. A leave‐one‐out sensitivity analysis was conducted to assess the influence of individual studies on the overall pooled estimate. All statistical analyses were conducted in RStudio (Version 764) using the “meta” and “metafor” packages.

## 3. Results

A comprehensive literature search across multiple databases initially yielded 219 records. Following the removal of duplicates, 173 unique articles remained for title and abstract screening. Based on predefined eligibility criteria, 9 studies were selected for full‐text review, resulting in the inclusion of 5 articles [19–23]. To complement the database search, a snowballing strategy was performed using the reference lists of the included studies and three additional reviews on the same topic [8, 24, 25], identifying 192 additional records. After screening these, four additional studies met the inclusion criteria [26–29]. The complete study selection process, including reasons for exclusion, is detailed in Figure 1.

**Figure FIGURE 1:**
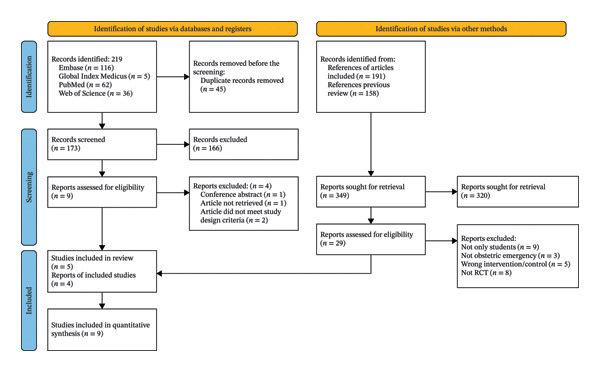
Flowchart of included studies.

### 3.1. Characteristics of the Studies

A total of nine randomized controlled trials were included, comprising 572 participants who evaluated educational interventions aimed at improving skills for the management of obstetric emergencies among students and healthcare professionals. The studies were conducted across different continents, including Turkey, Hong Kong, the United States, Iran, India, and Portugal, and were published between 2004 and 2025, reflecting broad geographical and temporal diversity [19–23, 26–29].

Most studies involved academic populations in the health sciences, particularly nursing, midwifery, and medical students, as well as obstetrics and gynecology residents. A smaller proportion included practicing midwives. Sample sizes varied considerably across studies, ranging from 20 to 107 participants, with relatively balanced distributions between intervention and control groups.

Regarding the type of obstetric emergency addressed, the most frequently investigated topics were eclampsia [19, 23, 26, 29], postpartum hemorrhage [20–22], and shoulder dystocia [27, 28]. This distribution underscores the focus on major causes of acute maternal morbidity and mortality, reinforcing the clinical relevance of the interventions analyzed.

Intervention groups received structured training or active educational strategies focused on the practical management of simulated obstetric emergencies, while control groups followed traditional teaching methods or standard practice without the use of simulation or specific reinforcement. All studies adopted a randomized controlled design comparing active training methods with conventional educational approaches.

The study populations were predominantly composed of young women, reflecting the academic profile of the participants. Reported mean ages ranged from 20.77 ± 1.22 years among nursing students [23] to 25.30 ± 5.17 years among midwifery students [21]. In the study by Mangla et al. [19], which included undergraduate medical students, the mean age was 22.18 ± 1.46 years in the intervention group and 21.89 ± 1.20 years in the control group. Other studies did not report age.

Regarding sex distribution, most participants were female, consistent with the demographic profile of health‐related academic programs. In three studies, 100% of the participants were women [21, 23, 28]. Mangla et al. [19] were the only study with a mixed sample, comprising 58% men and 42% women in the intervention group, and 52.6% men and 47.4% women in the control group. In the remaining studies, demographic data on age and sex were not reported.

Overall, the included studies were conducted in controlled academic environments, with moderate sample sizes and a focus on the acquisition and retention of practical skills in obstetric simulation scenarios addressing highly relevant clinical emergencies. Despite some heterogeneity in sample composition and reporting of demographic data, methodological consistency was observed regarding participants’ educational level and intervention setting. Further details on the included studies are provided in Table 1.

**Table TABLE 1:** The main characteristic of the included studies.

Authors/year	Country	Study design	Population included	Obstetric emergency	Number of participants		Gender^†^		Mean age (years)^∗^	
					Intervention	Control	Intervention	Control	Intervention	Control
Akalin and Sahin (2019)	Turkey	Open label	Nursing students	Eclampsia	53	54	13.2/86.8	13.0/87.0	20.77 ± 1.22	20.77 ± 1.22
Chan et al. (2025)	Hong Kong	Open label	Midwifery students	Postpartum hemorrhage	45	53	0/100	0/100	—	—
Deering et al. (2004)	United States	Single‐blind	Obstetric and gynecologic residents	Shoulder dystocia	16	17	—	—	—	—
Fisher et al. (2010)	United States	Open label	Obstetric and gynecologic residents	Eclampsia	25	13	—	—	—	—
Magee et al. (2013)	United States	Open label	Family medicine residents	Eclampsia and postpartum hemorrhage	10	10	—	—	—	—
Reynold et al. (2010)	Portugal	Open label	Midwives	Shoulder dystocia	26	24	0/100	0/100	—	—
Kordi et al. (2016)	Iran	Open label	Midwifery students	Postpartum hemorrhage	35	65	0/100	0/100	24.42 ± 4.44	25.30 ± 5.17
Mangla et al. (2024)	India	Open label	Undergraduate medical students	Eclampsia	24	19	58.0/42.0	52.6/47.4	22.18 ± 1.46	21.89 ± 1.20
Karadas and Terzioglu (2019)	Turkey	Open label	Students of the nursing faculty	Postpartum hemorrhage	73	10	—	—	—	—

^∗^Mean ± standard deviation.

^†^Men/women (%).

### 3.2. Meta‐Analysis of Included Studies

#### 3.2.1. Post‐Test Knowledge

Seven studies were included in the meta‐analysis, comprising a total of 333 healthcare students [19–21, 23, 27–29]. Akalin and Sahin [23] assessed knowledge acquisition using the KAF, Kordi et al. [21] evaluated applied cognitive performance through the Scores of Estimation of Postpartum Hemorrhage Volume (SEPHV), and five additional studies employed self‐developed questionnaires to measure post‐test knowledge immediately after the intervention [19, 20, 27–29]. The pooled analysis demonstrated a positive association favoring simulation‐based methods (SMD = 0.84; 95%CI 0.36 to 1.32; PI −0.36 to 2.05; *p* = 0.0005; *I* ^2^ = 75.1%) (Figure 2). This corresponded to an approximate estimated difference of +8.4 points (95% CI 3.6 to 13.2) in favor of simulation‐based learning. Because this estimate is derived from a standardized effect size, it should be interpreted as an approximate translation rather than a direct pooled mean difference. However, the PI crossed the null effect, indicating that the effect of simulation‐based education in a future comparable setting may range from absent or trivial to large. Sensitivity analysis did not alter the results (Figure 3). Visual inspection of the funnel plot indicated possible publication bias due to evident asymmetry (Figure 4).

**Figure FIGURE 2:**
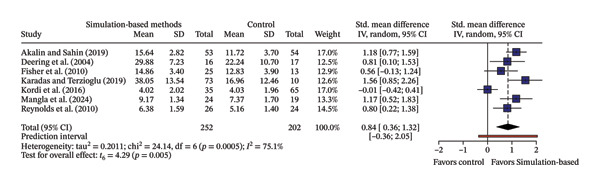
Forest plot of the meta‐analysis comparing post‐test knowledge scores between simulation‐based education and conventional teaching methods.

**Figure FIGURE 3:**
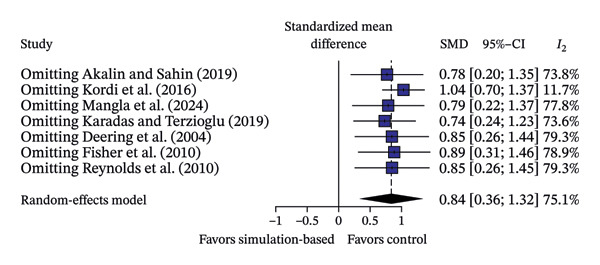
Leave‐one‐out sensitivity analysis of post‐test knowledge outcomes.

**Figure FIGURE 4:**
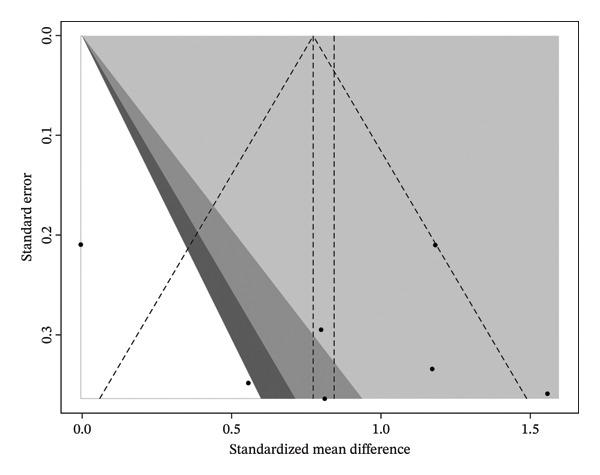
Contour‐enhanced funnel plot for the meta‐analysis of post‐test knowledge outcomes. Each point represents an individual study, plotted according to its standardized mean difference (*x*‐axis) and standard error (*y*‐axis), with studies at the top being more precise. The vertical dashed line indicates the pooled effect estimate, and the diagonal dashed lines represent the pseudo 95% confidence limits around the pooled estimate. The shaded regions denote contours of statistical significance relative to the null effect, with darker shading indicating areas of greater statistical significance. Visual asymmetry in the distribution of studies suggests the possibility of publication bias or small‐study effects.

#### 3.2.2. Teamwork

Chan et al. [22] evaluated teamwork outcomes using two instruments: the Self‐Efficacy of Teamwork Competencies Scale (SFETCS) and the Clinical Teamwork Scale (CTS). Both groups received theoretical instruction on postpartum hemorrhage, while the intervention group additionally participated in a structured Crew Resource Management‐based Simulation Training (CRM‐ST) program. Regarding self‐efficacy in teamwork, both groups demonstrated significant within‐group improvements over time; however, between‐group differences reached statistical significance only at 8 weeks postintervention (mean difference = 0.268 ± 0.131, *p* = 0.044), favoring the intervention group. Teamwork performance, assessed objectively with the CTS across five domains (communication, situational awareness, decision‐making, role responsibility, and patient friendliness), also showed significantly higher mean scores in the intervention group compared with the control group for overall performance (*p* = 0.021), communication (*p* = 0.036), situational awareness (*p* = 0.028), and role responsibility (*p* = 0.029). No significant between‐group differences were observed for decision‐making or patient friendliness.

#### 3.2.3. Critical Thinking

Akalin and Sahin [23] evaluated critical thinking disposition using the California Critical Thinking Disposition Inventory (CCTDI), which measures multiple subdimensions including truth‐seeking, open‐mindedness, analyticity, systematicity, self‐confidence, and inquisitiveness. Both the experimental and control groups demonstrated similar baseline scores across all subscales, indicating no significant pretest differences. Following the intervention, the experimental group (trained through simulation‐based education) showed statistically significant improvements in several subdomains, particularly analyticity (*p* = 0.019) and inquisitiveness (*p* = 0.001), as well as in the total CCTDI score (233.12 ± 22.07 vs. 217.61 ± 29.79, *p* = 0.004), compared with the control group.

### 3.3. Risk of Bias of the Included Studies

In Domain 1 (bias arising from the randomization process), most studies were judged as presenting some concerns due to incomplete reporting of allocation concealment or unclear details regarding sequence generation [19, 23, 26, 27]. The remaining trials adequately described randomization and were rated as low risk. In Domain 2 (bias due to deviations from intended interventions), all studies were classified as low risk, as the interventions were well defined, and participants were consistently allocated and analyzed according to their assigned groups. In Domain 3 (bias due to missing outcome data), every study was judged as low risk because outcome data were complete or missingness was minimal and balanced between groups. In Domain 4 (bias in measurement of the outcome), all studies were rated as low risk. In Domain 5 (bias in selection of the reported result), most studies were evaluated as presenting some concerns since none had preregistered protocols or statistical analysis plans publicly available, limiting confirmation of selective outcome reporting. Only Mert Karadas and Terzioglu [20] were rated as low risk in this domain. The detailed assessment of the risk of bias for each study is summarized in Figure 5.

**Figure FIGURE 5:**
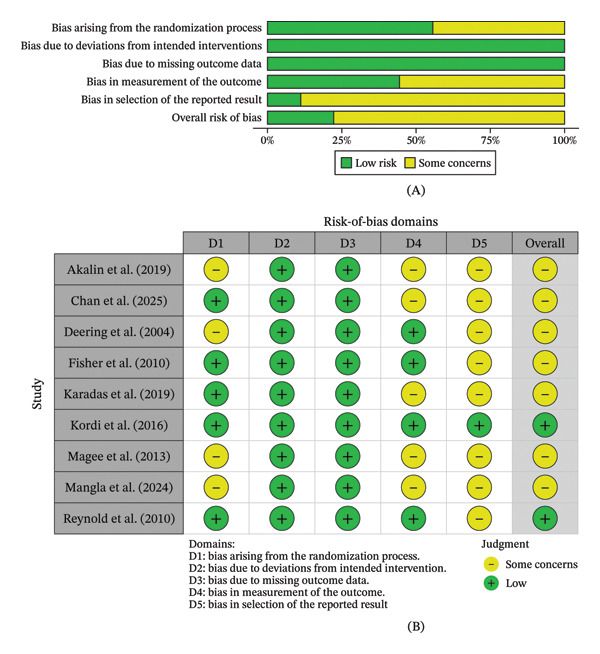
Risk of bias of included studies. (A) Summary plot of bias analysis. (B) Traffic light plot of bias analysis.

## 4. Discussion

Our meta‐analysis showed a moderate average improvement favoring simulation on post‐test knowledge, with complementary evidence of gains in teamwork (CRM‐based training improving CTS domains) and in critical thinking among students exposed to obstetric simulation. Notably, the wide PI indicates that future studies conducted in different settings could attenuate or even reverse the observed average effect, underscoring the need to interpret pooled effects alongside PIs [30].

We found no published minimal clinically important difference (MCID) specifically established for the KAF in obstetric education contexts. The back‐transformation to the KAF scale was intended to improve the interpretability of the SMD and should be viewed as an approximate estimate rather than a direct mean difference. In the absence of anchor‐based thresholds, distribution‐based rules suggest that ∼0.5 SD represents a meaningful change on cognitive scales, providing a pragmatic benchmark to judge whether KAF improvements are educationally important [31]. Applying this convention to typical KAF SDs aligns with our translated gain as likely meaningful for learners, while encouraging future studies to predefine anchor‐based MCIDs. Our findings reinforce prior reviews, showing that obstetric simulation improves technical skills, communication, and team performance, and extend the literature by offering updated, student‐focused quantitative estimates [25]. The literature has documented benefits in team training and hybrid/high‐fidelity approaches, consistent with our observed effects in knowledge and ancillary domains (teamwork, critical thinking) [6, 32].

In addition, the wide PI that we observed reflects substantial between‐study variance and cautions that the true effect in a new setting may plausibly range from trivial to large [33]. This finding supports a context‐dependent interpretation of simulation effectiveness rather than a uniform superiority across all educational settings. In our review, heterogeneity was likely influenced by differences in simulation fidelity and dose, debriefing structure, learner seniority, type of obstetric emergency, and outcome measurement. For example, undergraduate students and residents may respond differently to simulation because of differences in baseline clinical experience, role familiarity, and prior exposure to emergencies. Similarly, eclampsia, postpartum hemorrhage, and shoulder dystocia require distinct combinations of cognitive, technical, and nontechnical skills, while the included studies assessed outcomes using different instruments, ranging from written post‐test knowledge questionnaires to performance‐based measures.

Although only students were included in this meta‐analysis, populations and comparators still vary meaningfully, ranging from obstetrics and gynecology residents who were tested in high‐stakes emergency scenarios to undergraduate learners who were assessed on knowledge acquisition. Resident trials reported faster and more competent performance after simulation in a standardized shoulder dystocia scenario, with markedly shorter head‐to‐body delivery times among trained residents compared with untrained peers [27]. Undergraduate medical students, in contrast, were taught eclampsia management and showed larger gains in written post‐test scores with simulation than with conventional teaching, reflecting a different outcome construct than time‐critical technical performance [19]. Midwifery students estimating postpartum hemorrhage volume improved after training in all arms, yet there was no between‐group difference across web‐based, simulation‐based, and lecture formats, which again targets a different skill domain and may dilute pooled effects [21]. Nursing students learning preeclampsia management demonstrated gains in knowledge and critical thinking after high‐fidelity simulation, a profile that aligns more with cognitive outcomes than timed crisis performance [23].

Simulation modality, instructional dose, and scenario design also differ across studies and plausibly modulate effects. The eclampsia module for undergraduates used a high‐fidelity birthing simulator in two ninety‐minute sessions, with a standardized manual, checklists, and a structured curriculum, and emphasized both pre‐ and post‐testing within 1 month, which increases instructional intensity and standardization [19]. In the postpartum hemorrhage estimation trial, simulation consisted of a single four‐hour clinical skills center session, and the web‐based group engaged in 1 week of online study, both mapped to the same content outline, which may compress modality differences and reduce between‐group contrasts [21]. A factorial study in nursing students compared skills lab, standardized patients, and high‐fidelity simulation in multiple combinations, a design that enhances internal comparisons but introduces heterogeneity in exposure levels across arms and may amplify variance in pooled estimates [20]. Among residents, adding lecture to simulation did not yield incremental benefit relative to simulation alone for eclampsia management scores, which suggests diminishing returns from blended formats and may explain smaller effects in studies that combined methods [29].

Furthermore, the pattern observed by Chan et al. [22] in gain on teamwork outcomes aligns with prior syntheses, showing that team training improves teamwork processes and performance in healthcare settings, including communication and coordination, although the evidence is largely derived from practicing professionals rather than students [34]. In obstetrics specifically, a comprehensive review of multiprofessional simulation‐based programs reported improvements in team behaviors and suggested potential benefits for clinical outcomes in labor and delivery units, again focusing on health professionals and not learners in pre‐licensure programs [25]. Complementing this, a systematic review and meta‐analysis centered on obstetric emergency team training found associations with better patient outcomes at the unit level, which reflects effects in real‐world professional teams and a different target population than the student cohorts assessed in our synthesis [35].

Taken together, these prior meta‐analytic findings support the plausibility of the teamwork gains detected in the CRM‐based program described by Chan et al. [22], while underscoring an important distinction in population and implementation context. Our estimates pertain to healthcare students who are developing foundational competencies, whereas the referenced meta‐analyses evaluate trained professionals working within established clinical teams and organizational routines. This difference in baseline expertise, role clarity, and exposure to authentic clinical workflows likely contributes to variation in the magnitude and durability of teamwork improvements across studies.

Akalin and Sahin [23] reported improvements in critical thinking disposition among nursing students exposed to simulation‐based education, with gains in the total CCTDI and in selected subdomains compared with controls, consistent with an effect on attitudes that support analytic appraisal and inquiry. These findings are consistent with the idea that immersive scenarios, structured feedback, and guided reflection can cultivate dispositions that underpin clinical reasoning rather than only short‐term knowledge gains [23].

It is important to note that our review excluded studies that reported only subjective data on students’ experience, since this evidence was deemed outside the scope of the present synthesis. However, students reported outcomes in obstetric emergency simulations consistently describe high perceived value. In an undergraduate eclampsia trial, learners rated simulation as more interactive, more lifelike, and more motivating for self‐directed learning than traditional instruction, complementing superior test performance with favorable perceptions of relevance and engagement [19]. Qualitative work with midwifery students learning postpartum hemorrhage management describes perceived benefits for integrating theory with practice, gaining confidence under pressure, and clarifying roles during emergencies, highlighting affective and experiential gains that are not fully captured by written tests [36].

Similar signals appear across diverse student cohorts. A pilot of a postpartum hemorrhage scenario in prelicensure programs found improvements in knowledge accompanied by higher confidence and satisfaction, suggesting that realism and hands‐on rehearsal are drivers of positive appraisal [37]. A study of student–nurse midwives in Tanzania reported increases in self‐confidence and satisfaction after a focused postpartum hemorrhage simulation curriculum, reinforcing the generalizability of positive learner perceptions in resource‐constrained settings [38]. Earlier descriptive work in obstetrics education also noted that students value high‐fidelity simulation for the opportunity to apply concepts safely and to receive immediate feedback, which they perceive as accelerating readiness for real clinical encounters [39].

Beyond the quantitative findings, the present synthesis can be interpreted through the lens of educational theory. Simulation aligns with Kolb’s experiential learning theory, which emphasizes learning as a cyclical process involving active experimentation, concrete experience, reflective observation, and abstract conceptualization [40]. Immersive obstetric scenarios promote deeper cognitive engagement by linking theoretical content to experiential practice and guided reflection, mechanisms that explain the observed improvements in knowledge and critical thinking. From an evaluative perspective, these findings correspond to the second level of Kirkpatrick’s training evaluation model while also suggesting potential for higher levels, such as behavioral change and clinical results, that remain underexplored in prelicensure education [41]. From a curricular standpoint, the evidence underscores the pedagogical value of incorporating structured simulation into undergraduate and postgraduate healthcare training. Institutions may benefit from hybrid models that integrate theoretical sessions, hands‐on simulation, and structured debriefing. The debriefing phase, in particular, appears to be a critical determinant of learning transfer, fostering metacognitive awareness and reinforcing teamwork and communication [42].

### 4.1. Study Limitations

This review presents some limitations that should be considered when interpreting the findings. First, although the literature search was comprehensive and included multiple databases, publication bias cannot be ruled out, as suggested by the funnel plot asymmetry. Studies with negative or nonsignificant results may have been less likely to be published, potentially overestimating the pooled effect size. Second, the included trials exhibited considerable methodological heterogeneity regarding simulation fidelity, duration and frequency of training sessions, learner seniority, type of obstetric emergency, and assessment instruments used to measure learning outcomes. Third, the majority of studies assessed immediate postintervention knowledge rather than long‐term retention or transfer of skills to clinical practice. Therefore, the durability and real‐world impact of simulation‐based learning remain uncertain. Similarly, most studies focused on cognitive performance, whereas evaluations of technical and nontechnical skills (such as communication, leadership, and teamwork) were less frequent and based on small samples. Finally, the meta‐analysis was based on a relatively small number of randomized controlled trials with modest sample sizes, which restricts the precision and generalizability of the pooled estimates.

## 5. Conclusion

Simulation‐based education was associated with greater knowledge acquisition than traditional teaching methods in obstetric emergency training among healthcare students. However, substantial heterogeneity and PIs that cross the null suggest that the magnitude of the benefit may vary across contexts. These findings support the cautious integration of structured simulation into healthcare curricula. Future studies should use standardized assessment tools and evaluate long‐term retention, transfer to clinical practice, and maternal and neonatal outcomes.

## Author Contributions

Conceptualization: L.L.A.S.

Data curation: L.L.A.S., E.P.F., G.S.M.N., V.L.C., N.F.S., and L.T.M.

Methodology: L.L.A.S., V.R.S.S., L.S.B.S.O., and R.L.B.

Writing–original draft: L.L.A.S., E.S.R.B., V.R.S.S., E.P.F., G.S.M.N., L.S.B.S.O., V.H.O.R., and M.A.O.

Supervision: R.L.B.

Writing–review and editing: L.L.A.S., E.S.R.B., V.R.S.S., E.P.F., and G.S.M.N.

## Funding

The authors declare that no funds, grants, or other support were received during the preparation of this manuscript.

## Conflicts of Interest

The authors declare no conflicts of interest.

## Supporting Information

Additional supporting information can be found online in the Supporting Information section.

## Supporting information


**Supporting Information** Search Strategy.

## Data Availability

The data that support the findings of this study are available in the supporting information of this article.
