# Supplementary material for: Effectiveness of Simulation‐Based Teaching Methods in Learning Obstetric Emergencies Among Healthcare Students: A Systematic Review and Meta‐Analysis
Source: Obstet Gynecol Int. 2026 Jun 9;2026:7517075. doi: 10.1155/ogi/7517075 (PMC13250381; doi:10.1155/ogi/7517075)
Supplement: Supplementary file 1 — Supporting Information Search Strategy. [file OGI-2026-7517075-s001.docx]

**SUPPLEMENTARY MATERIAL 1 – SEARCH STRATEGY**

**Last review in databases:** September 15, 2025

**PubMed:**

("Students, Health Occupations"[Mesh] OR "Students, Medical"[Mesh] OR "Students, Nursing"[Mesh] OR "health occupation student*"[TIAB] OR "medical student*"[TIAB] OR "medicine student*"[TIAB] OR "nursing student*"[TIAB] OR "student nurse*"[TIAB] OR "midwifery student*"[TIAB] OR "student midwife*"[TIAB] OR "Education, Medical, Undergraduate"[Mesh] OR "health sciences student*"[TIAB] OR "nursing trainees"[TIAB] OR "undergraduate medical education"[TIAB] OR "undergraduate health education"[TIAB] OR "Clinical Clerkship"[Mesh] OR "Clinical Clerkship"[TIAB]) AND ("Pregnancy Complications"[Mesh] OR "pregnancy complications"[TIAB] OR "Hypertension, Pregnancy-Induced"[Mesh] OR "hypertension, pregnancy-induced"[TIAB] OR "Eclampsia"[Mesh] OR "eclampsia"[TIAB] OR "Pre-Eclampsia"[Mesh] OR "pre-eclampsia"[TIAB] OR "HELLP Syndrome"[Mesh] OR "HELLP syndrome"[TIAB] OR "Placenta Diseases"[Mesh] OR "placenta diseases"[TIAB] OR "Abruptio Placentae"[Mesh] OR "abruptio placentae"[TIAB] OR "Chorioamnionitis"[Mesh] OR "chorioamnionitis"[TIAB] OR "Fetal Diseases"[Mesh] OR "fetal diseases"[TIAB] OR "Meconium Aspiration Syndrome"[Mesh] OR "meconium aspiration"[TIAB] OR "Postpartum Hemorrhage"[Mesh] OR "postpartum hemorrhage"[TIAB] OR "Dystocia"[Mesh] OR "dystocia"[TIAB] OR "Abortion, Spontaneous"[Mesh] OR "abortion, spontaneous"[TIAB] OR "Abortion, Threatened"[Mesh] OR "abortion, threatened"[TIAB] OR "obstetric emergenc*"[TIAB] OR "Shoulder dystocia"[TIAB] OR "Uterine rupture"[TIAB] OR "Cord prolapse"[TIAB] OR "Amniotic fluid embolism"[TIAB] OR "Emergency obstetric care"[TIAB]) AND ("Simulation Training"[Mesh] OR "simulation training"[TIAB] OR "high fidelity simulation"[TIAB] OR "high-fidelity simulation"[TIAB] OR "patient simulation"[TIAB] OR "simulation-based education"[TIAB] OR "simulation-based learning"[TIAB] OR "simulated patient"[TIAB] OR "standardized patient"[TIAB] OR "manikin-based simulation"[TIAB] OR "objective structured clinical examination"[TIAB] OR "OSCE"[TIAB])

**Embase:**

('health student'/exp OR 'health student'/syn OR 'medical student'/exp OR 'medical student':ab,ti,kw OR 'medicine student':ab,ti,kw OR 'nursing student'/exp OR 'nursing student':ab,ti,kw OR 'student nurse':ab,ti,kw OR 'health sciences student':ab,ti,kw OR 'clinical clerkship'/exp OR 'clinical clerkship':ab,ti,kw OR 'midwifery student'/syn OR 'midwifery student':ab,ti,kw) AND ('pregnancy complication'/exp OR 'pregnancy complications'/syn OR 'maternal hypertension'/syn OR 'maternal hypertension'/exp OR 'eclampsia and preeclampsia'/syn OR 'hellp syndrome'/syn OR 'hellp syndrome':ab,ti,kw OR 'placenta disorder'/exp OR 'placenta disorder':ab,ti,kw OR 'premature rupture of membranes'/syn OR 'premature rupture of membranes':ab,ti,kw OR 'meconium aspiration'/syn OR 'obstetric hemorrhage'/syn OR 'obstetric hemorrhage':ab,ti,kw OR 'labor complication'/syn OR 'labor complication'/exp OR 'dystocia':ab,ti,kw OR 'abortion'/exp OR 'abortion'/syn OR 'obstetric emergency'/syn) AND ('simulation training'/exp OR 'simulation training'/syn OR 'simulation training':ab,ti,kw OR 'high fidelity simulation':ab,ti,kw OR 'high fidelity simulation training':ab,ti,kw OR 'patient simulation'/exp OR 'patient simulation'/syn OR 'patient simulation':ab,ti,kw)

**Global Index Medicus:**

("medical students" OR "nursing students" OR "medicine students" OR "student nurse*" OR "midwifery student*" OR "student midwife* OR "health sciences students" OR "undergraduate medical education" OR "undergraduate health education" OR "clinical clerkship") AND ("pregnancy complications" OR "maternal hypertension" OR "eclampsia" OR "pre-eclampsia" OR "HELLP syndrome" OR "placenta disorders" OR "abruptio placentae" OR "premature rupture of membranes" OR "chorioamnionitis" OR "meconium aspiration" OR "postpartum hemorrhage" OR "obstetric hemorrhage" OR "dystocia" OR "shoulder dystocia" OR "uterine rupture" OR "cord prolapse" OR

"amniotic fluid embolism" OR "threatened abortion" OR "spontaneous abortion" OR

"obstetric emergency" OR "emergency obstetric care") AND ("simulation training" OR "high fidelity simulation" OR "simulation-based education" OR "patient simulation" OR "simulated patient" OR "standardized patient" OR "manikin-based simulation" OR "objective structured clinical examination" OR "OSCE")

**Web of Science:**

('simulation training'/exp OR 'simulation training'/syn OR 'simulation training':ab,ti,kw OR 'high fidelity simulation':ab,ti,kw OR 'high fidelity simulation training':ab,ti,kw OR 'patient simulation'/exp OR 'patient simulation'/syn OR 'patient simulation':ab,ti,kw) AND

(TS=(

"pregnancy complications" OR "maternal hypertension" OR "eclampsia" OR "pre-eclampsia" OR

"HELLP syndrome" OR "placenta disorder" OR "abruptio placentae" OR

"premature rupture of membranes" OR "chorioamnionitis" OR

"fetal diseases" OR "meconium aspiration" OR

"postpartum hemorrhage" OR "obstetric hemorrhage" OR

"dystocia" OR "shoulder dystocia" OR "uterine rupture" OR

"cord prolapse" OR "amniotic fluid embolism" OR

"threatened abortion" OR "spontaneous abortion" OR

"obstetric emergency" OR "emergency obstetric care"

))

AND

(TS=(

"simulation training" OR "high fidelity simulation" OR "high-fidelity simulation training" OR

"patient simulation" OR "simulation-based education" OR "simulation-based learning" OR

"simulated patient" OR "standardized patient" OR "manikin-based simulation" OR

"objective structured clinical examination" OR "OSCE"

))
